# Common juniper, an overlooked conifer with high invasion potential in protected areas of Patagonia

**DOI:** 10.1038/s41598-023-37023-1

**Published:** 2023-06-17

**Authors:** Jorgelina Franzese, Ramiro Rubén Ripa

**Affiliations:** 1 Investigaciones de Ecología en Ambientes Antropizados, Instituto de Investigaciones en Biodiversidad y Medioambiente (CONICET-UNCo), R8400 S. C. Bariloche, Argentina; 2 Grupo de Genética Ecológica, Instituto de Investigaciones en Biodiversidad y Medioambiente (CONICET-UNCo), Evolutiva y de la Conservación, R8400 S. C. Bariloche, Argentina; 3 Instituto de Evolución, Ecología Histórica y Ambiente (CONICET-UTN), San Rafael, Mendoza Argentina

**Keywords:** Ecology, Plant sciences

## Abstract

The benefits of early detection of biological invasions are widely recognized, especially for protected areas (PAs). However, research on incipient invasive plant species is scarce compared to species with a recognized history of invasion. Here, we characterized the invasion status of the non-native conifer *Juniperus communis* in PAs and interface areas of Andean Patagonia, Argentina. We mapped its distribution and described both the invasion and the environments this species inhabits through field studies, a literature review, and a citizen science initiative. We also modeled the species’ potential distribution by comparing the climatic characteristics of its native range with those of the introduced ranges studied. The results show that *J. communis* is now widely distributed in the region, occurring naturally in diverse habitats, and frequently within and close to PAs. This species can be considered an incipient invader with a high potential for expansion in its regional distribution range, largely due to its high reproductive potential and the high habitat suitability of this environment. Early detection of a plant invasion affords a valuable opportunity to inform citizens of the potential risks to high conservation value ecosystems before the invader is perceived as a natural component of the landscape.

## Introduction

Protected areas (PAs) worldwide are recognized as a key component of the broad response to the environmental degradation caused by global change, mainly because of their crucial role in conserving biodiversity^1,2^. Paradoxically, these areas are suffering increased degradation due to processes related to global change, biological invasions being one of the most important drivers associated with this phenomenon^3–5^. In particular, the biodiversity and integrity of several PAs around the world are being jeopardized by the invasion of introduced plants^1^, a process fostered especially by human activity^6,7^. Since PAs are not entirely excluded from the major threats to biodiversity, the unique biological reservoirs contained in these areas are being increasingly compromised.

Most PAs are interspersed with or adjacent to a mosaic of landscapes altered by human influence^3,8^. The spatial configuration of these landscapes can facilitate a network of potential pathways for introduced species^9^. Indeed, the abundance and composition of non-native plant species in PAs are strongly influenced by their surroundings, mainly due to the rapid colonization of these species from belt zones^10^. Non-native plant species are undesirable in PAs, and those which are invasive are considered a priority for research and management^10–12^. In this regard there is abundant research on plant species with a recognized history of invasion and conspicuous impacts on natural areas (e.g. ref.^13,14^; however, research on incipient invasive species is relatively scarce (e.g. ref.^15^ and references therein). This is despite the ecological knowledge that incipient plant invaders may respond to efficient management strategies before they advance in the invasion process^16^ and have a significant ecological impact, at which point their eradication becomes unlikely^17^.

Although the benefits of early detection of incipient invasion in natural habitats are well recognized, so are the difficulties associated with it^18–20^. Detection of early invasion foci is usually fortuitous^16^, and citizen collaboration is important in increasing the probability of registering these situations. Public engagement is being enhanced by collaborative projects, led by professional scientists, that seek to compile information on potentially invasive species^20,21^. In particular, citizen science has emerged as a powerful tool for detecting and mapping the distribution of recent invasive species and obtaining diverse bio-ecological information on them^20,22,23^. This knowledge can provide insights into the invasion stage, the mechanisms behind the invasion, and the invader’s potential ecological impact, which can be context-dependent^24,25^.

Climate is recognized as the single most important factor determining the distribution of plant species at a large scale^26,27^. Thus, a frequently used approach to predict where a species might invade is analysis of the climatic similarity between its native range and areas outside it^28,29^, even for plant species with no invasive history^30^. This approach has been used for invasion risk assessment of non-native conifer species in areas of their introduced ranges throughout the Southern Hemisphere^28,30^, where they pose a significant threat to the diversity and functioning of native ecosystems^31^ and even PAs^32,33^. In particular, climate matching can be a valuable tool for estimating suitable areas for potentially damaging non-native conifers with incipient invasion. By cross-referencing information, it is possible to prioritize the search for and control of new invasion foci in, for example, PAs with high invasion risk.

The PAs of Andean Patagonia are no exception in terms of their high vulnerability to an increasing number of introduced plant species^34,35^. In this region, increasing anthropogenic pressure on PAs acts as a catalyst for new invasions of introduced plant species whose invasive status, ecology, impact, and distribution are mostly unknown. This is exemplified by the conifer *Juniperus communis* L. (native to temperate regions of the boreal hemisphere), which has been identified as a potential high-risk invader of climatically suitable areas in Africa^30^ and Oceania^28^. At the southernmost tip of South America, Argentina, *J. communis* has recently been officially cataloged as an invasive species (Ministerio de Ambiente y Desarrollo Sostenible 2021). Despite this, no studies have addressed its invasion status, distribution, or potential expansion range, especially in the areas of the country where it may represent a risk to biodiversity, such as the PAs. This species already has three validated records in PAs of Andean Patagonia, according to the Biodiversity Information System which provides biological information on the species, and PAs of Argentina (www.sib.gob.ar). However, *J. communis* can be frequently seen in PAs of Andean Patagonia, which suggests that it is under-recorded, probably because of its incipient invasion (i.e. the earliest stage of the invasion process). This assumption of an incipient invasion is supported by the lack of *J. communis* registers in key reference literature describing the regional flora^36^, including literature focusing on introduced plant species in the main PAs of the region^35,37^. In addition, this species is increasingly valued as the raw material for producing gin, an alcoholic beverage that is booming internationally. This encourages its cultivation in the area, which can increase the source of propagules for invasions in nearby PAs. It can also be seen in gardens; however, its incidence as an ornamental plant, and therefore the importance of this type of use as a source of propagules, is as yet unknown.

Here, we characterized the invasion status of the non-native conifer *J. communis* in PAs and interface areas of Andean Patagonia, Argentina, by mapping its distribution and describing both the invasion and the environments this species inhabits. We registered the type of invaded habitats, species abundance, its spatial configuration pattern, the accompanying woody species, the species’ reproductive potential (i.e. presence of reproductive plants and seedlings), its importance as an ornamental plant, and its occurrence in PAs and associated areas. We also modeled the potential distribution of the species by comparing the climatic conditions in its introduced range in Patagonia with those of its native distribution range. We used different methodological approaches to acquire data on the species in the region: a literature search, field sampling, and citizen collaboration. To our knowledge, this is the first work to provide information on *J. communis* as an invader of a South American country. We address key descriptive aspects of the current *J. communis* invasion that provide clues to the ecological mechanisms involved in its spread. Knowledge of the potential distribution of *J. communis* could be useful in determining the invasion risk the species presents for high conservation value ecosystems of Patagonia.

## Results

### *Juniperus communis *in Andean Patagonia

**Figure 1:**
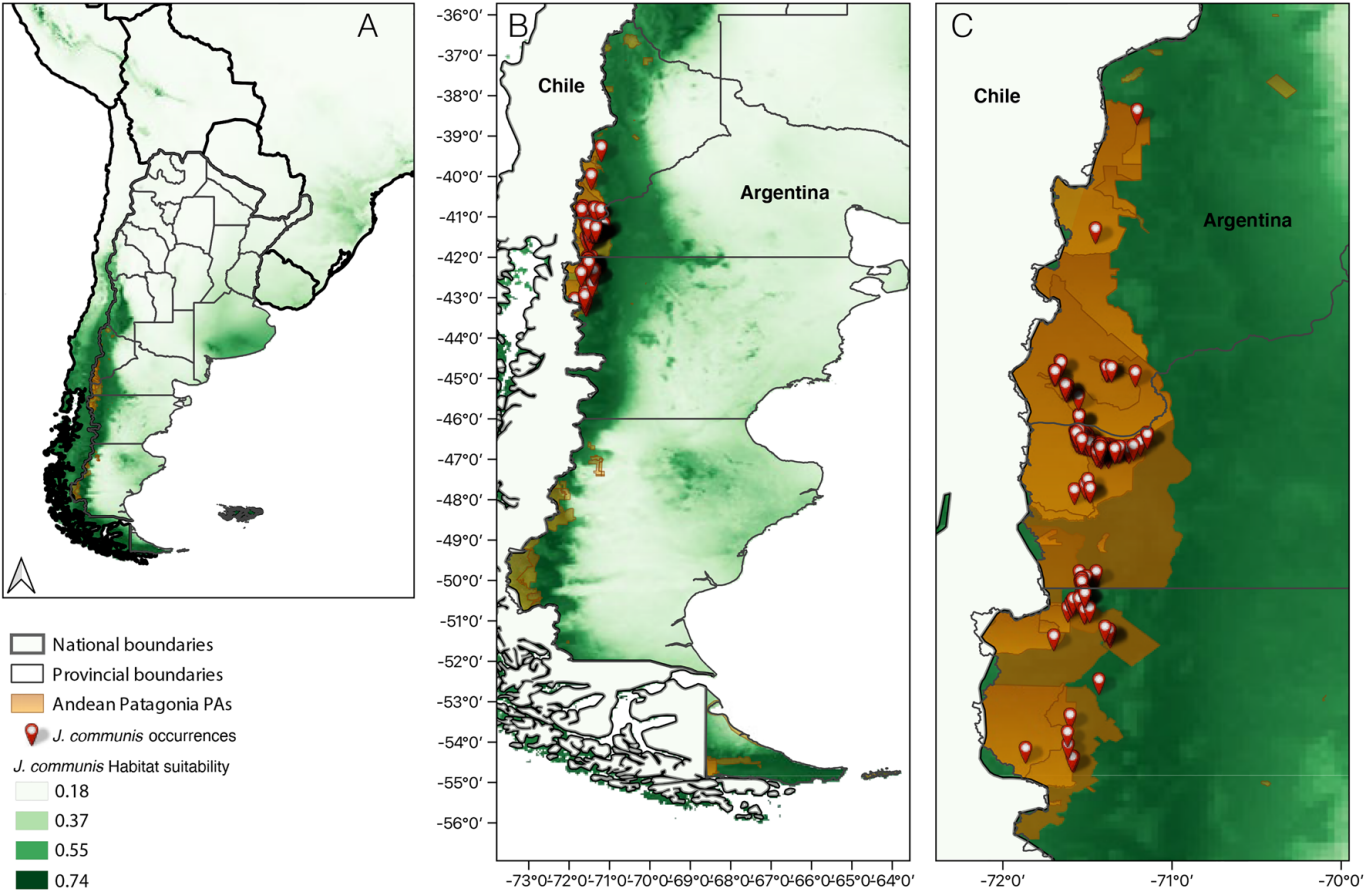
*Juniperus communis* occurrences (red symbols) in PAs and urban-natural interface areas (orange area) of Andean Patagonia, Argentina panels (**B**) and (**C**). In the three panels the potential distribution model for *J. communis* generated by Maxent is shown, panel (**A**) shows a general view of Argentina and neighboring countries, panel (**B**) shows the entire Patagonia and panel (**C**) shows a zoom to the sampled area. Habitat suitability is represented on a green scale, with darker colors representing higher suitability. In (**C**), the largest lighter orange area represents the World Biosphere Reserve, which overlaps most of the other PAs. The map was created using QGIS version 3.28.2-Firenze (www.qgis.org).

**Figure 2:**
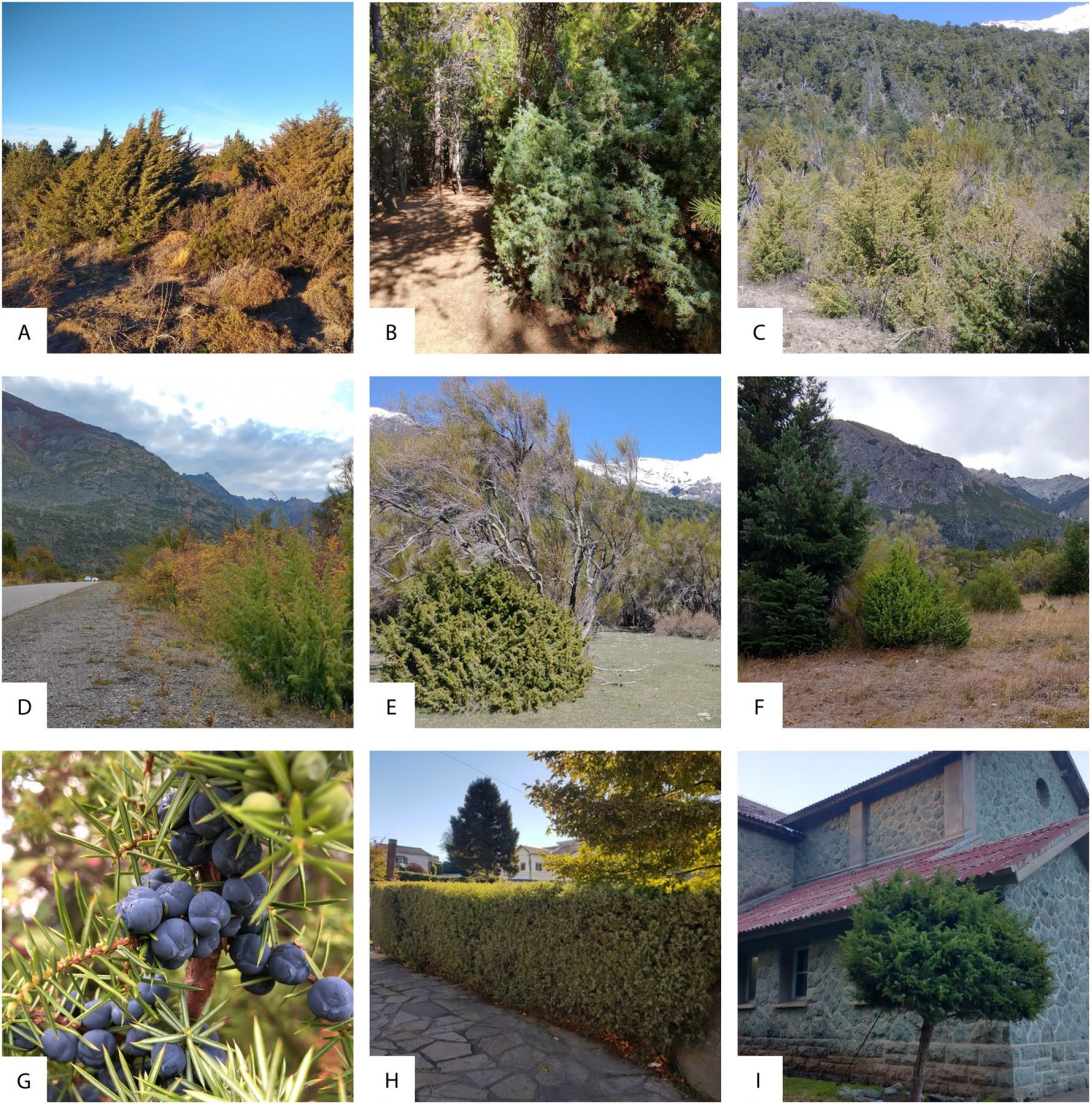
A. Steppe invasion, B. Forest, walking trail invasion, and C. High-abundance shrubland invasion. D. Roadside invasion. E. Co-occurrence with a native woody species (*Diostea juncea*). F. Co-occurrence with a non-native woody species (*Pinus contorta*). G. Mature (purple) and immature (green) fruits on the same individual. H. *Juniperus communis’ hedge. I. Ornamental tree*. The photographs in this research work were captured by the authors at diverse locations spanning the study area.

We compiled 174 occurrences of *J. communis* in the region (58.6% from field sampling, 33.9% from citizen contributions, and 7.4% from the literature review); > 90% of these records were from PAs (Fig. 1). We detected the presence of *J. communis* within eight PAs and close to another seven in the region (Table 1). Almost 100% of occurrences (sampled or reported) were associated with disturbed environments, mostly represented by roadsides (gravel or paved) and trails (Fig. 2).

**Table 1:** List of protected areas (PAs) in Andean Patagonia.

PAs of Andean Patagonia with high habitat suitability for *J. communis*
Área Natural Protegida Boca del Chimehuín
Área Natural Protegida Cipresal de las Guaitecas
Área Natural Protegida Cuchillo—Curá
**Área Natural Protegida Los Alerces**
Área Natural Protegida Provincial del Sistema Domuyo
Área Natural Protegida Río Azul—Lago Escondido
Monumento Natural Provincial Cañada Molina
Paisaje Protegido Río Limay
**Parque Nacional Lago Puelo**
**Parque Nacional Lanín**
Parque Nacional Los Arrayanes
Parque Nacional Los Glaciares
**Parque Nacional Nahuel Huapi**
Parque Nacional Patagonia
Parque Nacional Perito Moreno
Parque Provincial Azul
Parque Provincial Copahue—Caviahue
**Parque Provincial y Reserva Forestal Río Turbio**
**Parque y Reserva Nacional Los Alerces**
Parque y Reserva Provincial Península de Magallanes
**Reserva de Biósfera Andino Norpatagónica**
Reserva Forestal Batea Mahuida
Reserva Forestal Chañy
Reserva Forestal de Uso Múltiple Lago Epuyén
Reserva Nacional Lago Puelo
Reserva Nacional Lanín
Reserva Nacional Los Glaciares
**Reserva Nacional Nahuel Huapi**
Reserva Nacional Perito Moreno
Reserva Natural Silvestre El Rincón
Reserva Natural Silvestre La Ascensión
Reserva Natural Silvestre Patagonia
Reserva Natural Silvestre Piedra del Fraile
Reserva Natural Turística Nant y Fall (Arroyo Las Caídas)
Reserva Natural Turística Piedra Parada
Reserva Provincial Lago Baggilt
Reserva Provincial Lago del Desierto
Reserva Provincial Lagunas de Epulafquen
Reserva Provincial Punta Gruesa
Reserva Provincial San Lorenzo
Reserva Provincial Tucu—Tucu
Sitio RAMSAR y Parque Provincial del Tromen
Sitio RAMSAR, Parque y Reserva Nacional Laguna Blanca
Parque Nacional Tierra del Fuego
Reserva Corazón de la Isla
Reserva Cultural y Natural Playa Larga
Reserva Natural Silvestre Isla de los Estados y Archipiélago de Año Nuevo
Reserva Provincial de Uso Múltiple Laguna Negra
Reserva Provincial de Uso Múltiple Río Valdez
Sitio Ramsar Glaciar Vinciguerra y Turberas Asociadas
Sitio RAMSAR y Reserva Hemisférica Costa Atlántica de Tierra del Fuego

Names in bold show PAs with *J. communis* presence, while underlined names show PAs with *J. communis* registered in their surroundings. Our study classified all PAs as having high habitat suitability (> 0.74) for *J. communis.*

**Figure 3:**
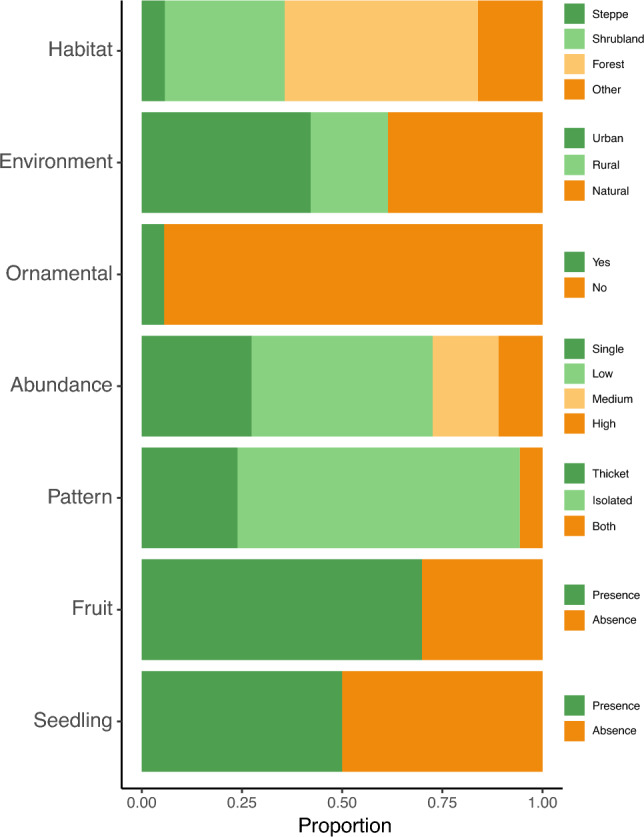
Descriptive variables related to *J. communis* and the environments it inhabits. The graph depicts the proportion of registers for different sub-categories according to habitat (steppe, shrubland, forest, other; n = 87), environment (natural, rural, urban; n = 114) if the species was used as ornamental (n = 161), species abundance (single, low, medium, and high; n = 73), the spatial configuration pattern of the individuals (thicket, isolated, both; n = 88), and the presence of fruits (n = 103) and seedlings (n = 72).

**Figure 4:**
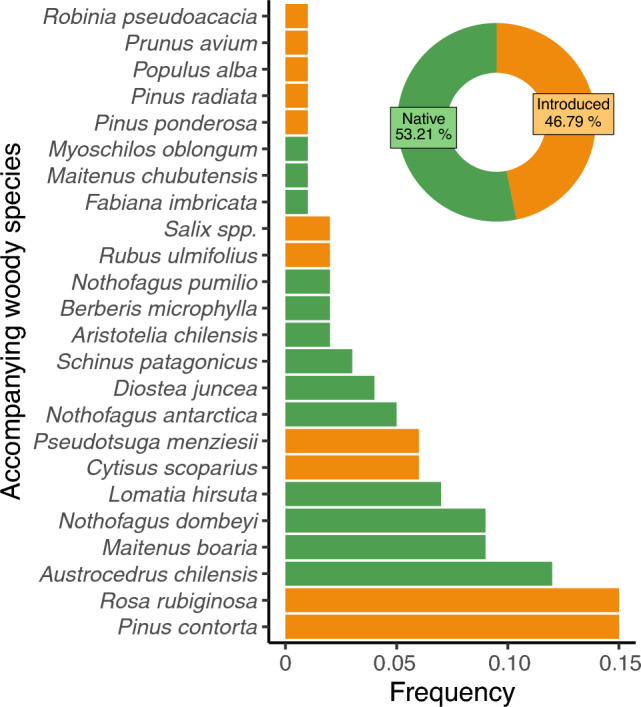
Frequency of the principal woody species that accompanied *J. communis* occurrences (n = 38). The X-axis represents the proportion of sites where each species was observed, with a maximum value of 0.15 indicating that these species were found in 15% of the sampled sites. Bar colors indicate species origin: native (green) or introduced (orange). The donut figure shows the percentage of occurrences for native and introduced species.

Field sampling data indicated that *J. communis* was found most frequently in forests (48%), followed by shrublands (26%), with the lowest representation in steppe environments (5%; Fig. 2). We found an equal occurrence of the species in natural and urban habitats, with only a small percentage of ornamental use (Figs. 2, 3). Regarding the spatial distribution pattern, *J. communis* was frequently found as isolated individuals (62%), followed by thickets (21%) and, to a lesser extent, both patterns in the same site (5%; Figs. 2, 3). The species was found mostly at low abundance (2–10 individuals in 45% of the occurrences), followed by a single individual (24%), medium density (11–100 individuals in 16% of the occurrences), and high density (> 100 individuals in 11% of the occurrences; Figs. 2, 3). Fruited individuals and seedlings were observed in ca. 70%, and 50% of the registers, respectively (Fig. 3), which could represent an underestimation of seedling presence since the understory of some sites was difficult to explore due to dense vegetation. In addition, we registered 24 main woody species accompanying *J. communis*, half of which were native (Fig. 4). The most frequently found native species were *Austrocedrus chilensis* (Cupressaceae), *Maytenus boaria* (Celastraceae), *Nothofagus dombeyi* (Nothofagaceae), and *Lomatia hirsuta* (Proteaceae). Among the non-native species the most frequently found were *Pinus contorta* (Pinaceae), *Rosa rubiginosa* (Rosaceae), and *Cytisus scoparius* (Fabaceae) (Figs. 1, 4).

The literature review afforded 18 records of *J. communis* cited as a naturally established species in the Andean Patagonian region (Neuquén, Río Negro, and Chubut provinces), with the oldest record dating back to 2002 (Table 2). Most of the records corresponded to PAs (82%), including four national parks. In most of the studies the inclusion of *J. communis* was not intentional, rather it appeared when describing vegetation, or when listing introduced species (Table 2). Only three studies considered the species as the focus of their research, although none of these recognized it as invasive (Table 2).

**Table 2:** Published literature (listed by year) that refers to *J. communis* established in natural communities of Patagonia, Argentina.

Year	#	Focus	Recognized status	Reason for inclusion	PA	Location	Author/s
2022	1	No	None	Vegetation description	No	Bariloche	Masciocchi et al.^38^
2021	2	Yes	Non-native	Test of endozoochory seed dispersal by a native marsupial	Yes	*NHNP*	Vazquez et al*.*^39^
		No	Exotic	Vegetation description	Yes	*NHNP*	Moguilevsky et al*.*^40^
2019	1	No	Exotic	Vegetation description	Yes	*NHNP*	NHNP management plan^41^
2018	1	No	Introduced	Vegetation description	Yes	*NHNP*	Martín-Albarracín et al*.*^42^
2017	1	Yes	Exotic	Register of length, growth and architecture	No	Las Golondrinas	Stecconi et al*.*^43^
2015	3	No	Exotic	Vegetation description	Yes	*NHNP*	Blackhall et al*.*^44^
		No	Exotic	Detection of exotic species	Yes	*LAPN*	Kutschker et al*.*^45^
		No	Invasive	Record of richness and abundance of exotic species with fleshy fruits	Yes	*PMLL* (RN)	Iglesias^46^
2014	1	No	Introduced	Description of invasion pattern of Pinaceae	Yes	*NHNP*	Relva & Nuñez^47^
2013	1	No	Exotic	Update of list of species for 4 national parks	Yes	*NHNP, LANP, LNP, LPNP*	Brion et al*.*^48^
2012	1	No	Exotic	Identification of plants with medicinal value used by settlers	Yes	*LANP*	Toledo & Kutschker^49^
2009	1	No	Exotic	Identification of exotic species, for control purposes	Yes	*LPNAP*	Rovere et al*.*^50^
2008	1	Yes	Endemic to the NH	Determination of botanical and chemical characteristics	?	Chubut	Guerra et al*.*^51^
2006	1	No	Exotic	Vegetation description	No	Bariloche	Dzendoletas et al*.*^52^
2005	1	No	Adventitious	List of vascular plant flora of NHNP	Yes	Neuquén, Río Negro	Ezcurra & Brion^34^
2003	1	No	Introduced	Vegetation description	Yes	*NHNP*	Simberloff et al*.*^53^
2002	1	No	Introduced	Record of abundance and status of introduced woody species	Yes	*NHNP*	Simberloff et al*.*^54^

*PA* protected area, *LNP* Lanín National Park, *LPNP* Lago Puelo National Park, *LANP* Los Alerces National Park, *NHNP* Nahuel Huapi National Park, *PMLL* Parque Municipal Llao-llao. ?: not provided. *NH* north hemisphere.

### Potential distribution and bioclimatic matching

**Figure 5:**
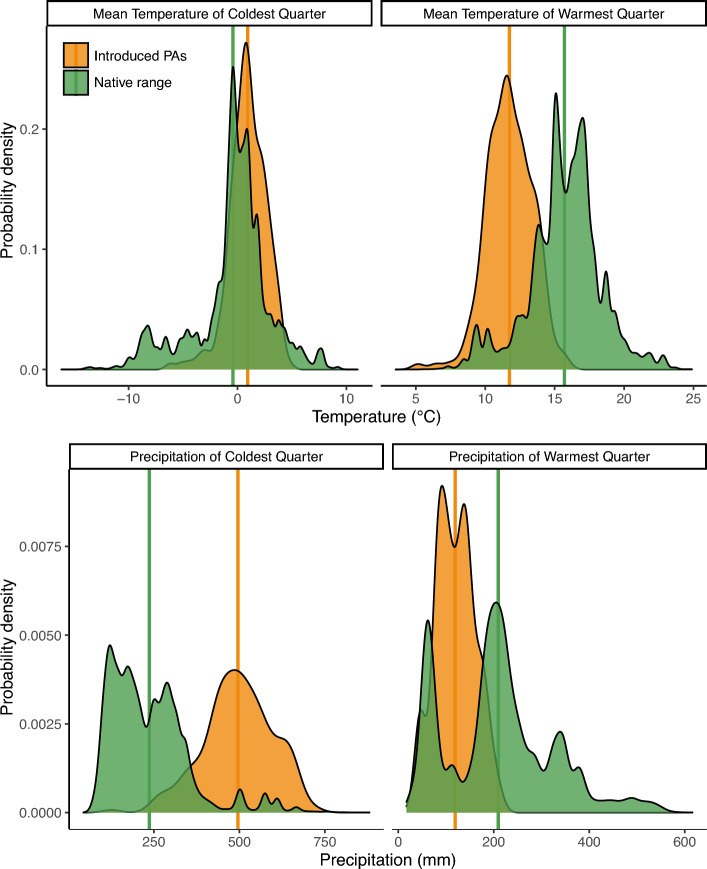
Comparison of environmental variables between the PAs of Patagonia and the native range of *J. communis*: mean temperature of coldest and warmest quarters (top panel), and mean precipitation of coldest and warmest quarters (bottom panel). The Y-axis represents the estimated probability density. In all cases a P-value < 0.001 was observed for comparisons by using the Anderson–Darling test. Mean values for the environmental variables are represented as vertical lines for the native range (green) and the introduced areas (orange).

The Andean Patagonian region showed a highly climatically suitable land area for *J. communis* occurrence (Figs. 1, 5), with the area of the best fitting model covering all major PAs in the region (Fig. 1). The area of greatest suitability occupies the region near the Andes, from central to southern Argentina, becoming longitudinally wider towards the north of Andean Patagonia and extending eastward into the southern part of Río Negro and northern Chubut.

The three environmental variables of major importance to the *J. communis* distribution model were the mean temperature of the warmest and coldest quarters and the precipitation of the coldest quarter (Table S1). The functional relationship between the four continuous predictor variables studied and the predicted habitat suitability (Fig. S1), shows that the highest values for probability of presence are given for a mean temperature of the warmest quarter between 10 and 20 °C, a mean temperature of the coldest quarter between − 20 and 10 °C and precipitation of the coldest quarter superior to 200 mm (Fig. S1). The bioclimatic variables analyzed for the PAs of Andean Patagonia differed from those of the native range of *J. communis*: PAs in Andean Patagonia showed higher mean temperatures and precipitation in the coldest quarter and lower mean temperature and precipitation in the warmest quarter than the species’ native range (Fig. 5). These results were supported by the jackknife test (Fig. S2), which also showed that the mean temperature of the coldest quarter had the most useful information when considered alone (highest gain in isolation), and information that was not present in the other variables (highest gain decrease when omitted).

## Discussion

*Juniperus communis* has been in the Andean Patagonian region for at least 90 years, and the results of our work show that the time lag between its introduction and its invasion is coming to an end. Records of an intentional introduction of this species in northwestern Patagonia date back to the 1930s, when it was to be cultivated for ornamental purposes^55^. Seventy years later the species was registered in the same location, but occurring naturally^56^. Our results indicate that *J. communis* has achieved a wide distribution in Andean Patagonia, occurring naturally in diverse habitats, with numerous occurrences inside and close to PAs. The information we gather in this study allows us to characterize *J. communis* as an incipient invader with high potential for expansion of its regional distribution range. The likelihood of this spread can be largely determined by the high reproductive potential of the species and high habitat suitability of the invaded region.

The wide distribution of *J. communis* in the region can be partially explained by seed dispersal, which probably occurs via endozoochory by common species of the regional fauna. The main dispersers of *J. communis* in its native range are birds of the genus *Turdus*, which is also represented in Andean Patagonia. In other regions, for example, in England, *T. viscivorus*, *T. merula*, and *T. philomelos* have been identified as the main dispersers of *J. communis* seeds^57^, while in mountainous regions of the European Mediterranean the seeds of this species are dispersed almost exclusively by *T. torquatus* and *T. viscivorus*^58,59^. In Andean Patagonia, the genus *Turdus* is mainly represented by *T. falcklandii* (www.sib.gob.ar)^60^, which consumes fleshy fruits of common shrub species^61,62^, including those of *J. communis* (Lambertucci S., pers. comm.). Additionally, there are records of apparently viable seeds of this species in the feces of hares^63^ and red deer (Relva A., pers. comm.). It is interesting to note that *T. falcklandii* is the most important frugivore present during winter^61^. Unlike most functionally equivalent native woody species, during winter *J. communis* bears fruits, which may represent a reproductive advantage for the invader^64^. The preliminary evidence described for this incipient invader highlights the importance of studying aspects of its reproductive ecology (e.g. phenology) that may provide clues to mechanisms that facilitate its spread and its potential impact on the recipient communities.

Although dispersal is an important factor in determining plant species’ spread, climatic conditions are decisive in determining establishment success. The results showed high habitat suitability for the species based on climate variables, which was evidenced in the high proportion of the sampled sites with seedlings and fruit-bearing individuals; that is, plant stages indicative of population growth. This contrasts with what is currently happening to *J. communis* in different areas of its native range, where the number of populations and their size have decreased drastically, mainly due to a lack of natural recruitment^65,66^. This is mainly associated with a low percentage of viable seeds^67^, which could be due in part to climate effects. For example, Garcia et al.^65^ demonstrated that rainy spring periods (short but heavy storms) in open shrublands of the Mediterranean mountains negatively affected the viability of *J. communis* seeds by impeding pollen dispersal. This effect is probably not as pronounced in the areas of incipient invasion in Patagonia, where rainfall is scarce during the period of pollen dispersal (i.e. the warmest quarter of the year) and is even lower than that registered in the species’ native range (Fig. 5). Furthermore, in Patagonia, the scarcity of rainfall during the warmest period of the year is expected to be accentuated by climate change in the coming years^68,69^. The disparities in climatic variables between native and introduced ranges could indicate adaptation^70^ or phenotypic plasticity^71^. This highlights the importance of closely monitoring species like *J. communis*, to evaluate a potential climatic niche shift^72,73^, and to reassess these invasions in prospective climate change scenarios. Many other effects may be involved in *J. communis* pollination failure in particular^57,74^, and in its population decline in general in areas of its native range^57,67^. Concerning the latter, the incidence of a recently invasive pathogen in Europe, the oomycete *Phytophthora austrocedri*, is causing widespread mortality in *J. communis* native populations^75^. This pathogen is already present in Andean Patagonia^76,77^, so it would be interesting to evaluate its incidence in the introduced populations of the woody invader, as well as the incidence of other factors that negatively affect the species in its native range but seem not to hinder its expansion in Patagonia.

Belt zones control the number of invaders in PAs, determining the entry and spread of these species into the natural vegetation matrix^7,9^. *Juniperus communis* was associated with roads and walking trails that were in close contact with natural vegetation, which was evidenced by the high proportion of native woody species that accompanied it*.* Among the accompanying species were trees characteristic of Andean-Patagonian forests, such as *Austrocedrus chilensis* and *Nothofagus dombeyi*. In turn, the high frequency of these tree species reflects the prevalence of *J. communis* in forest ecosystems. Among the most frequently cited non-native species there were long-time invaders, highly adapted to human-modified environments, such as *Pinus contorta* and *Rosa rubiginosa* (Fig. 4; www.sib.gob.ar). Considering that forest habitats are suitable for *J. communis* invasion, and that disturbed areas represent expansion opportunities for this species, the increased degradation caused by new trails (that deviate from those officially delimited) produced by domestic animals and visitors to PAs^78^ is of great concern. On the other hand, although medium to high abundance invasion was observed in ca. one-fourth of the sampled sites, it was common to find individuals in small groups or alone (i.e. isolated from other conspecifics but not from other woody species). While individuals established far from parent plants may indicate an increase in the spatial occupancy of the species, it also reveals that conditions for its control in areas of conservation concern may be favorable (i.e. small population size^15,79^). As pointed out for other conifer invasive species^80^, the relatively low growth rate of woody plants affords a time window during which on-the-ground action can be taken before the incipient invasion takes hold – even a single plant can constitute a significant propagule source to the surroundings^15^.

Unlike other woody plants that became invaders of natural environments associated with urban areas^81,82^, *J. communis* was infrequently found as an ornamental or a living fence plant. Therefore, the current use of this species does not represent a major threat in terms of invasion spread. However, attention should be paid to other human-induced propagule sources. In light of the increasing valuation of this species for gin production, it would be interesting to investigate the importance of emerging cropping areas as a source of propagules that could spread to natural areas, as well as the generation of protocols to minimize its potential dispersal and consequent invasion risk. On the other hand, while fruit harvesting by local people from natural populations can reduce propagule pressure, it also favors positive public perception of the species as being of value as an economic resource^83^. Thus, the control of incipient invaders could be a particularly difficult challenge in areas associated with PAs, due to the cultural importance and economic value certain invasive species can represent for residents and visitors^84,85^.

## Conclusions

We present here the first documentation of the distribution and descriptive characteristics of an incipient invasion of *J. communis* in PAs of Andean Patagonia, Argentina. Although the results indicate that the species has high spreading potential, they also show that this is an opportune moment for its control in areas that merit conservation.

Since belt areas are important in mediating introduced plant biodiversity in PAs, raising citizen awareness of environmental issues such as plant invasion is crucial. Citizen science is a powerful tool when used as a means of informing and raising awareness of the consequences of individual actions (e.g. selection of ornamental garden species) when living beside or close to natural areas. Awareness of the potential impact of introduced non-native species in natural-urban interfaces can promote a greater demand for native species, which also has multiple advantages for both the user and the environment^86^. Scientists have an important role to play in achieving this goal; for example, by leading citizen science projects and promptly communicating their research results to the public, thus constructing a two-way process that should be strengthened over time. This process could be especially important in the case of incipient plant invasions since people can receive a timely warning about the potential risks of invasive species before they are perceived as a natural component of the landscape and become valued.

## Methods

### Study area

The abrupt longitudinal precipitation gradient, moisture availability and temperature of Andean Patagonian brings about a transition in vegetation from humid forests in the west to steppe environments in the east^87^. The study area is covered mainly by plants of the Subantarctic biogeographic province and, to a lesser extent, the High Andean and Patagonian biogeographic provinces^88^. The Subantarctic province is characterized by temperate and cold forests, both deciduous and evergreen, especially conifers and southern beeches of the genus *Nothofagus*; the High Andean province is characterized by a dominance of xerophytic grasses and creeping or cushion dicotyledons; and the Patagonian province is represented by ingressions of the Patagonian steppe with scattered low compact shrubs and abundant bare soil – the grasses found here are mainly low^88^.

In Andean Patagonia there are at least 51 PAs (Table 1) with different jurisdictions, zoning, and degrees of protection. Most PAs are intermingled in a mosaic with different types of land use. For example, one of the largest PAs, Parque Nacional Nahuel Huapi (710.000 ha), is spread over several municipalities whose urban fabric is in close contact with areas of natural vegetation. The majority of these municipalities are tourist areas (e.g. San Carlos de Bariloche, Villa La Angostura, San Martín de Los Andes), which leads to high connectivity with other urbanizations, increasing the likelihood of spreading introduced species and, therefore, generating incipient invasions.

### Study species

*Juniperus communis* L. (common juniper, enebro; Cupressaceae) grows as a shrub or upright tree (up to 12 m high) but can also acquire a prostrate form, presumably in response to environmental conditions^57^. The species is usually dioecious and reproduces predominantly by sexual means^89^. Rooting of decumbent branches occurs in areas with an oceanic climate although it is not clear whether these branches survive when the original shrub dies^57^. Female individuals produce axillary green globose strobiles, which turn bluish-black when mature^57^. Cones present unusually fleshy and fused scales that give it a berry-like appearance and take two to three years to mature^66^ (Fig. 1). Therefore, reproductive female plants can carry fruits at different stages of maturity all year round^57^. The native range of this species is Panarctic, occurring from the southern Arctic to about 30° latitude in North America, Europe, and Asia^90^. In terms of climate, *J. communis* occupies very different environments, with limitations due to cold (Arctic and Polar and Northern Urals), drought (Mediterranean and Southern Urals), or high soil moisture (Eastern Alps)^90^. Its growth rate is strongly controlled by temperature and limited by soil moisture^90^. Moreover, high temperatures can decrease its seed viability, particularly by disrupting the growth of the pollen tube and female gametophyte, as well as fertilization^91^. Since ancient times this species has been widely used for culinary, medicinal, and ornamental purposes^92,93^. In Andean Patagonia, local people harvest the fruits from natural populations and sell them to gin production companies located in the region and other parts of the country. There is a record of the species entering this region in the 1930s, when it was introduced along with other non-native conifers to be cultivated for ornamental purposes on Isla Victoria (northwestern Andean Patagonia)^55^.

### Sampling design—*Juniperus communis* in Andean Patagonia

To describe the invasion of *J. communis* we compiled records of its location (latitude and longitude) in PAs and their interface areas in the Andean Patagonian region (Argentina). Data were obtained through field surveys, a literature review, and the contributions of citizens. For each data source, a set of additional variables to describe the invasion and the environment were also recorded. The number and type of variables depended on the data source.

In autumn 2022 we carried out field surveys in an area that encompassed protected areas and their urban-natural interface areas (− 40.63, − 42.97; − 71.87, − 71.65). As we traveled along main and secondary roads (paved and gravel roads, respectively) and walking trails, we searched for *J. communis* individuals (sampling point). At each sampling point (with at least 1 km between points) we registered: species location, habitat type (steppe, shrubland, forest, or other), environment type (natural, rural, or urban), the abundance of individuals (single: 1, low: 2–10, medium: 11–100, and high: > 100), the spatial configuration of the individuals (thicket, isolated, or both), the main woody species, whether the species was occurring naturally or not (e.g. ornamental), and if there were individuals with fruits and seedlings (< 0.25 m^58^). The presence of fruits and seedlings was considered a proxy for reproductive potential.

In addition, to compile a set of data we reviewed the scientific literature and literature specializing in the regional flora (books and technical reports) that reported the occurrence (location) of *J. communis*. In May 2022 we searched the scientific literature on Scopus using the following terms: “Juniperus AND communis OR enebro OR juniper AND Patagonia AND Argentina”. The reference lists from the articles found were also searched for other relevant publications not found in the initial search. In addition, to know when and how many times the species has been registered as naturally occurring in the region, and how it was recognized in terms of invasive status, we looked for articles that reported *J. communis* as part of the natural vegetation; we registered: the publication year, whether the species was the focus of the article (i.e. if it was intentionally selected to be studied or not), its recognized status (e.g. introduced, invasive species), the reason why it was included in the study, and if it was registered in a protected area.

During autumn 2022 we also made a call to citizens through social networks, requesting data on the location of *J. communis*, along with information on how to identify it. We enabled a number of WhatsApp accounts for citizens to send their records to, since this application was one of the preferred ways for people in Argentina to report species sightings^23^. We asked citizens to give the species’ location, provide a picture of the plant to verify its identity, and report whether any observed plant had fruits. The fruiting plants could be easily detected by citizens; it is unlikely they would be confused with the similar fruits of common woody natives (i.e. species with small, rounded, purple fruits) whose fructification period ended in midsummer, before the survey period^64^. From both the citizen contributions and the literature review methods we obtained extra information on, for example, species abundance and habitat type. We incorporated these data into the database and indicated in the results section the number of records for each variable presented.

### Potential distribution and bioclimatic matching

To estimate the potential distribution of *J. communis* based on climatic parameters that define the habitat suitability of Andean Patagonia, Argentina, we constructed Species Distribution Models (SDMs) for each species using the Maxent software^94^, a maximum entropy modeling method that generates a continuous binomial probability distribution of habitat suitability. For this we used the records obtained through field sampling and citizen science (Table S2), and also records that we downloaded from the gbif database for *J. communis var. communis* updated to May 31, 2022. All available worldwide occurrences in gbif were considered; however, only occurrences within its native range were found. In total we obtained 133,981 records^95^, of which 40,263 were complete with geographic coordinates and were used for habitat suitability modeling. We used the 19 bioclimatic variables available in WORLDCLIM version 2.0 as environmental predictors for the model^96^. However, since many of Worldclim’s bioclimatic variables are highly correlated, to avoid errors generated by data multicollinearity, we chose 4 bioclimatic variables: precipitation of the coldest quarter, precipitation of the warmest quarter, mean temperature of the coldest quarter and mean temperature of the warmest quarter. Our selection criteria for the variables were based on Pearson's correlation analysis (r < 0.7)^97^ and on relevant bio-ecological knowledge of the species^90,91,98,99^. We used layers with a resolution of 2.5 min for these variables. The model was developed using 75% of the location data, while the remaining 25% was used to validate the model. The algorithm was run with 1000 iterations, through which MaxEnt increases the model gain by modifying the coefficient of a single feature as a function of the input environmental data. The accuracy of the model was tested using the area under the curve of the receiver operating characteristic (ROC)^100^. The contribution of each variable to the final model was determined by randomly permuting the values of that variable between training points (both presence and background) and measuring the resulting decrease in the area under the curve. Values were normalized to obtain percentages. The relative strength of each predictor variable was assessed using the Maxent Jackknife test of variable importance. This test shows the importance of the environmental variables by detecting (i) the variable with the greatest explanatory power, and (ii) the variable with the greatest amount of unique information (not contained in the other variables). Finally, to test whether the observed bioclimatic characteristics coincide between the PAs of the introduced area in Andean Patagonia (200 randomly selected sites) and records from the native range, we quantitatively compared the values of the 4 environmental variables included in the model. Values for the native range and the PAs of Andean Patagonia were extracted with the point sampling tool in Qgis and compared using the Anderson–Darling test. The QGIS version 2.18 spatial analysis software was applied to edit and process all the maps shown in this work. All the reported models, tests, and graphs were performed in R^101^.

### Ethical approval

The use of plant parts in the study complies with international, national, and institutional guidelines.

## Supplementary Information

Supplementary Information.

## Data Availability

The data used for the SDM, collected in the framework of this work, are available in the supplementary material (Table S2). All other data generated and/or analyzed during this study are available from the corresponding author on reasonable request.
