# Author Correction: Common juniper, an overlooked conifer with high invasion potential in protected areas of Patagonia

**DOI:** 10.1038/s41598-023-38496-w

**Published:** 2023-07-13

**Authors:** Jorgelina Franzese, Ramiro Rubén Ripa

**Affiliations:** 1 Investigaciones de Ecología en Ambientes Antropizados, Instituto de Investigaciones en Biodiversidad y Medioambiente (CONICET-UNCo), R8400 S. C. Bariloche, Argentina; 2 Grupo de Genética Ecológica, Instituto de Investigaciones en Biodiversidad y Medioambiente (CONICET-UNCo), Evolutiva y de la Conservación, R8400 S. C. Bariloche, Argentina; 3 Instituto de Evolución, Ecología Histórica y Ambiente (CONICET-UTN), San Rafael, Mendoza Argentina

Correction to: *Scientifc Reports* 10.1038/s41598-023-37023, published online 17 June 2023

The original version of this Article contained errors. In Figure 1b, ‘Chile’ was incorrectly located on the map. The original Figure 1 appears below.

**Figure 1:**
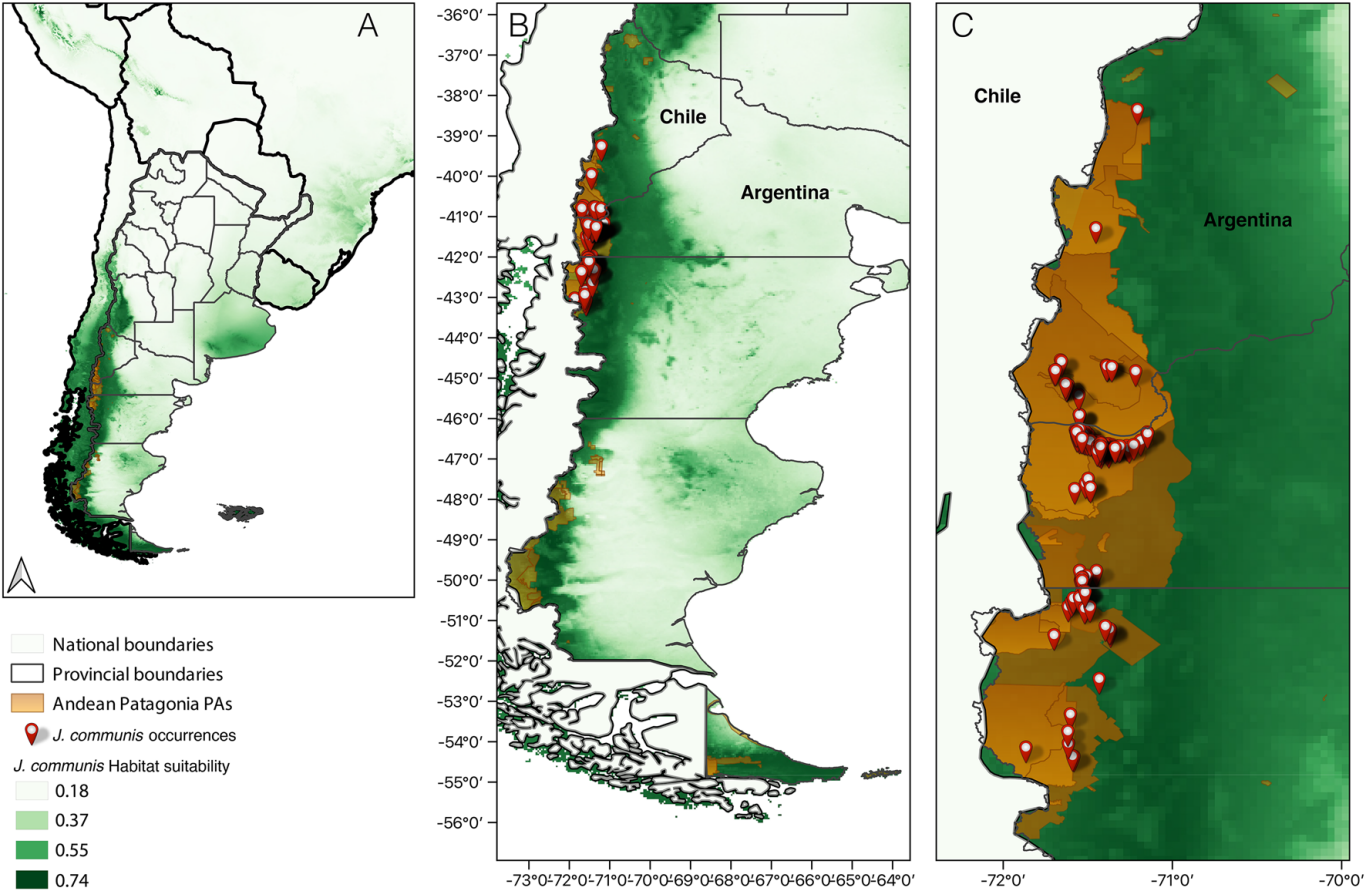
*Juniperus communis* occurrences (red symbols) in PAs and urban-natural interface areas (orange area) of Andean Patagonia, Argentina, panels (**B**) and (**C**). In the three panels the potential distribution model for *J. communis* generated by Maxent is shown, panel (**A**) shows a general view of Argentina and neighboring countries, panel (**B**) shows the entire Patagonia and panel (**C**) shows a zoom to the sampled area. Habitat suitability is represented on a green scale, with darker colors representing higher suitability. In (**C**), the largest lighter orange area represents the World Biosphere Reserve, which overlaps most of the other PAs. The map was created using QGIS version 3.28.2-Firenze (www.qgis.org).

Furthermore, the Figure legends of Figures 2 and 3, were switched.

The legend of Figure 2,

“Descriptive variables related to *J. communis* and the environments it inhabits. The graph depicts the proportion of registers for different sub-categories according to habitat (steppe, shrubland, forest, other; n = 87), environment (natural, rural, urban; n = 114), if the species was used as ornamental (n = 161), species abundance (single, low, medium, and high; n = 73), the spatial configuration pattern of the individuals (thicket, isolated, both; n = 88), and the presence of fruits (n = 103) and seedlings (n = 72). The photographs in this research work were captured by the authors at diverse locations spanning the study area.”

now reads:

“A. Steppe invasion, B. Forest, walking trail invasion, and C. High-abundance shrubland invasion. D. Roadside invasion. E. Co-occurrence with a native woody species (*Diostea juncea*). F. Co-occurrence with a non-native woody species (Pinus contorta). G. Mature (purple) and immature (green) fruits on the same individual. H. *Juniperus communis*’ hedge. I. Ornamental tree. The photographs in this research work were captured by the authors at diverse locations spanning the study area.

Additionally, the legend of Figure 3,

“A. Steppe invasion, B. Forest, walking trail invasion, and C. High-abundance shrubland invasion. D. Roadside invasion. E. Co-occurrence with a native woody species (*Diostea juncea*). F. Co-occurrence with a non-native woody species (*Pinus contorta*). G. Mature (purple) and immature (green) fruits on the same individual. H. *Juniperus communis*’ hedge. I. Ornamental tree.”

now reads:

“Descriptive variables related to *J. communis* and the environments it inhabits. The graph depicts the proportion of registers for different sub-categories according to habitat (steppe, shrubland, forest, other; n = 87), environment (natural, rural, urban; n = 114) if the species was used as ornamental (n = 161), species abundance (single, low, medium, and high; n = 73), the spatial configuration pattern of the individuals (thicket, isolated, both; n = 88), and the presence of fruits (n = 103) and seedlings (n = 72).

The original Article has been corrected.

