# Supplementary material for: Common juniper, an overlooked conifer with high invasion potential in protected areas of Patagonia
Source: Sci Rep. 2023 Jun 17;13:9818. doi: 10.1038/s41598-023-37023-1 (PMC10276858; doi:10.1038/s41598-023-37023-1)
Supplement: Supplementary file 1 — Supplementary Information. [file 41598_2023_37023_MOESM1_ESM.docx]

Supplementary information

**Common juniper, an overlooked conifer with high invasion potential in protected areas of Patagonia**

Jorgelina Franzese, Ramiro Rubén Ripa

**Table S1.** Estimates of relative contributions of the environmental variables to the Maxent model. To determine the first estimate, in each iteration of the training algorithm the increase in regularized gain is added to the contribution of the corresponding variable, or subtracted from it if the change to the absolute value of lambda is negative. For the second estimate, for each environmental variable in turn, the values of that variable on training presence and background data are randomly permuted. The model is reevaluated on the permuted data, and the resulting drop in training AUC is shown in the table, normalized to percentages.

| **Variable** | **Percent contribution** | **Permutation importance** |
| --- | --- | --- |
| Mean Temperature of Warmest Quarter (bio 10) | 2.6 | 27.6 |
| Mean Temperature of Coldest Quarter (bio 11) | 31.9 | 57.7 |
| Precipitation of Warmest Quarter  (bio 18) | 5.9 | 2.5 |
| Precipitation of Coldest Quarter  (bio 19) | 59.6 | 12.3 |

**Figure S1.** Response curves of how each environmental variable affects the Maxent prediction. (A) The curves show how the predicted probability of presence changes as each environmental variable is varied, keeping all other environmental variables at their average sample value. In other words, the curves show the marginal effect of changing exactly one variable, whereas the model may take advantage of sets of variables changing together. (B) Each curve represents a different model created using only the corresponding variable. These plots reflect the dependence of predicted suitability on both the selected variable and on dependencies induced by correlations between the selected variable and other variables.


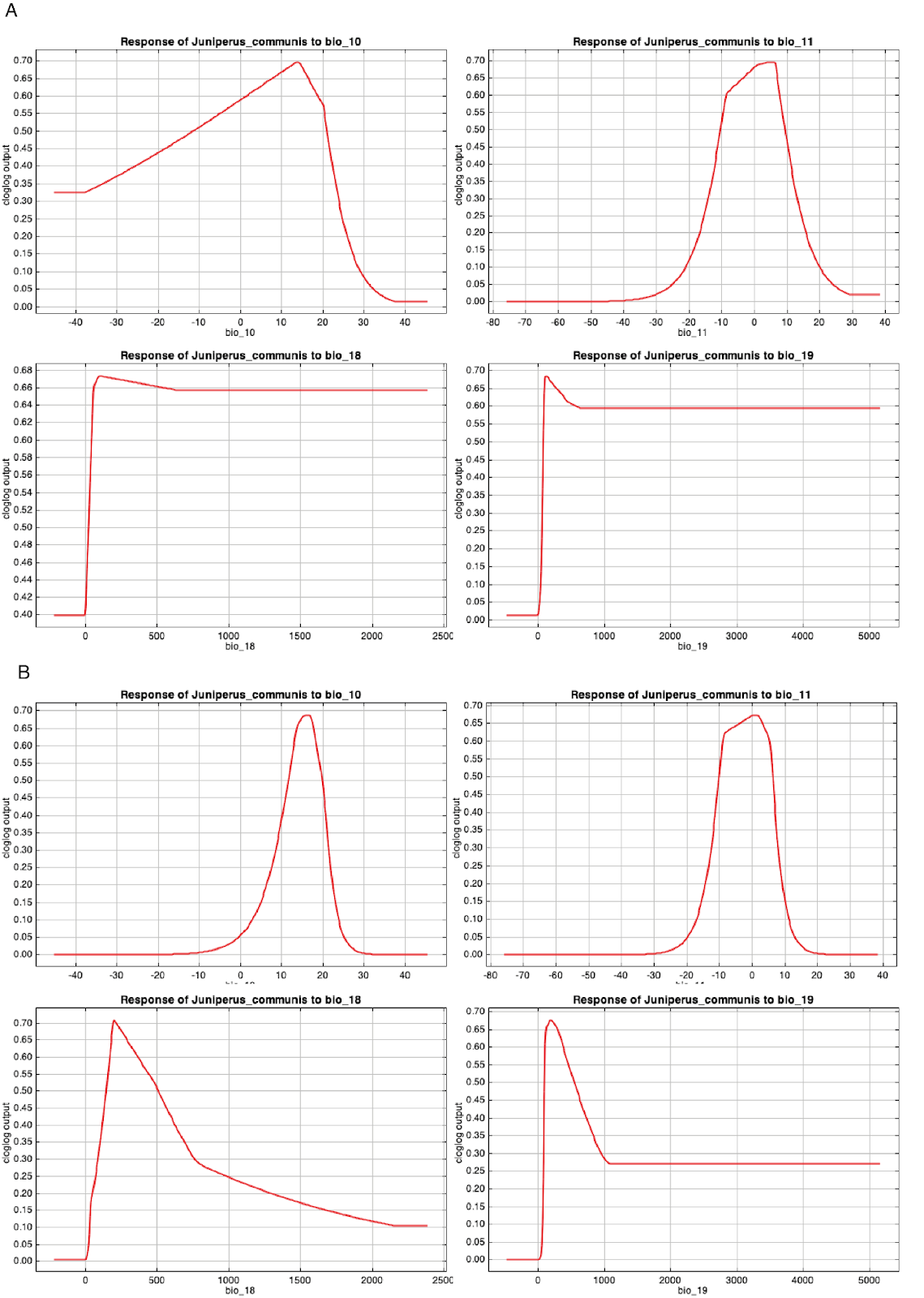


**Figure S2.** Jackknife test for the importance of each individual environmental variable in the development of the MaxEnt model relative to all the environmental variables (red bar) for each predictor variable alone (blue bars), and the drop in training gain when the variable is removed from the full model (light blue bars).


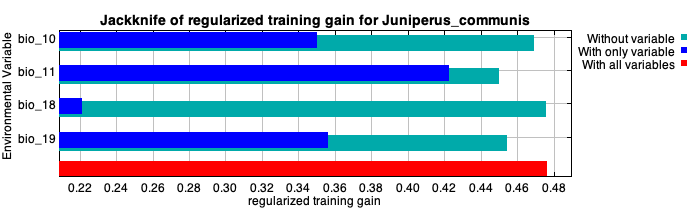


**Table S2.** Occurrence localities registered for *J. communis* in Patagonia. Records came from fieldwork, bibliography and Citizen Science.

| Source | Year | Latitude | Longitude | References |
| --- | --- | --- | --- | --- |
| Citizen Science | 2022 | -42.969869 | -71.866667 | This work. |
| Citizen Science | 2021 | -42.086006 | -71.616667 | This work. |
| Citizen Science | 2022 | -42.083333 | -71.615087 | This work. |
| Citizen Science | 2022 | -42.054614 | -71.603306 | This work. |
| Citizen Science | 2022 | -42.052052 | -71.596649 | This work. |
| Citizen Science | 2022 | -42.039868 | -71.59655 | This work. |
| Citizen Science | 2022 | -42.034525 | -71.591053 | This work. |
| Citizen Science | 2022 | -42.030287 | -71.587249 | This work. |
| Citizen Science | 2022 | -42.01976 | -71.585373 | This work. |
| Citizen Science | 2022 | -42.0061111 | -71.582813 | This work. |
| Citizen Science | 2022 | -42.006111 | -71.577778 | This work. |
| Citizen Science | 2022 | -41.9983333 | -71.574284 | This work. |
| Citizen Science | 2022 | -41.998318 | -71.569927 | This work. |
| Citizen Science | 2022 | -41.994385 | -71.56958 | This work. |
| Citizen Science | 2022 | -41.992682 | -71.568771 | This work. |
| Citizen Science | 2022 | -41.992225 | -71.5666667 | This work. |
| Citizen Science | 2022 | -41.9438418 | -71.565532 | This work. |
| Citizen Science | 2022 | -41.9314354 | -71.5617052 | This work. |
| Citizen Science | 2022 | -41.929463 | -71.553775 | This work. |
| Citizen Science | 2022 | -41.925492 | -71.550114 | This work. |
| Citizen Science | 2022 | -41.920512 | -71.55 | This work. |
| Citizen Science | 2022 | -41.916465 | -71.55 | This work. |
| Citizen Science | 2022 | -41.9146822 | -71.55 | This work. |
| Citizen Science | 2022 | -41.913384 | -71.55 | This work. |
| Citizen Science | 2022 | -41.9105 | -71.55 | This work. |
| Citizen Science | 2022 | -41.388056 | -71.545 | This work. |
| Citizen Science | 2022 | -41.379316 | -71.5440626 | This work. |
| Citizen Science | 2022 | -41.371667 | -71.541824 | This work. |
| Citizen Science | 2022 | -41.3708333 | -71.539986 | This work. |
| Citizen Science | 2022 | -41.3621164 | -71.539878 | This work. |
| Citizen Science | 2022 | -41.354853 | -71.5393451 | This work. |
| Citizen Science | 2022 | -41.327466 | -71.5383333 | This work. |
| Citizen Science | 2022 | -41.177139 | -71.538333 | This work. |
| Citizen Science | 2022 | -41.174404 | -71.53821 | This work. |
| Citizen Science | 2022 | -41.1685556 | -71.537025 | This work. |
| Citizen Science | 2022 | -41.1621111 | -71.535836 | This work. |
| Citizen Science | 2022 | -41.162110 | -71.5347222 | This work. |
| Citizen Science | 2022 | -41.157869 | -71.534129 | This work. |
| Citizen Science | 2022 | -41.1541389 | -71.533875 | This work. |
| Citizen Science | 2022 | -41.1510556 | -71.5330876 | This work. |
| Citizen Science | 2022 | -41.145124 | -71.531313 | This work. |
| Citizen Science | 2022 | -41.144775 | -71.523919 | This work. |
| Citizen Science | 2022 | -41.14455 | -71.522442 | This work. |
| Citizen Science | 2022 | -41.14145 | -71.520231 | This work. |
| Citizen Science | 2022 | -41.1413929 | -71.5198639 | This work. |
| Citizen Science | 2022 | -41.1401111 | -71.519487 | This work. |
| Citizen Science | 2022 | -41.13968 | -71.519051 | This work. |
| Citizen Science | 2022 | -41.139444 | -71.518179 | This work. |
| Citizen Science | 2022 | -41.1386944 | -71.516643 | This work. |
| Citizen Science | 2022 | -41.137196 | -71.516643 | This work. |
| Citizen Science | 2022 | -41.1370556 | -71.5166389 | This work. |
| Citizen Science | 2022 | -41.1368056 | -71.5144444 | This work. |
| Citizen Science | 2022 | -41.136055 | -71.51423 | This work. |
| Citizen Science | 2022 | -41.136 | -71.514028 | This work. |
| Citizen Science | 2022 | -41.1357778 | -71.507096 | This work. |
| Citizen Science | 2022 | -41.1355556 | -71.506819 | This work. |
| Literature | 2021 | -42.966813 | -71.696306 | Moguilevsky et al. 2021 |
| Literature | 2015 | -42.916667 | -71.692063 | Blackhall et al. 2015 |
| Literature | 2015 | -42.892701 | -71.691056 | Iglesias 2014 |
| Literature | 2012 | -42.82059 | -71.661833 | Toledo & Kutschker 2012 |
| Literature | 2009 | -42.719722 | -71.656639 | Rovere et al. 2009 |
| Literature | 2015 | -42.510806 | -71.631819 | Blackhall et al. 2015 |
| Literature | 2022 | -42.234883 | -71.627326 | Masciocchi et al. 2022 |
| Literature | 2018 | -42.218983 | -71.626339 | Martín-Albarracín et al. 2018 |
| Literature | 2017 | -42.216495 | -71.624643 | Stecconi et al. 2017 |
| Literature | 2015 | -42.213741 | -71.624232 | Kutschker et al. 2015 |
| Literature | 2014 | -42.199037 | -71.618911 | Relva & Nuñez 2014 |
| Literature | 2003 | -42.09488 | -71.618562 | Simberloff et al. 2003 |
| Literature | 2002 | -42.093228 | -71.617006 | Simberloff et al. 2002 |
| Sampled | 2022 | -41.901386 | -71.550 | This work. |
| Sampled | 2022 | -41.872617 | -71.5497147 | This work. |
| Sampled | 2022 | -41.872512 | -71.548767 | This work. |
| Sampled | 2022 | -41.1352822 | -71.504169 | This work. |
| Sampled | 2022 | -41.135282 | -71.503987 | This work. |
| Sampled | 2022 | -41.134972 | -71.499001 | This work. |
| Sampled | 2022 | -41.134966 | -71.498368 | This work. |
| Sampled | 2022 | -41.134335 | -71.4933333 | This work. |
| Sampled | 2022 | -41.133918 | -71.491111 | This work. |
| Sampled | 2022 | -41.1335924 | -71.489984 | This work. |
| Sampled | 2022 | -41.133215 | -71.488 | This work. |
| Sampled | 2022 | -41.132925 | -71.486351 | This work. |
| Sampled | 2022 | -41.131543 | -71.484995 | This work. |
| Sampled | 2022 | -41.1312222 | -71.467637 | This work. |
| Sampled | 2022 | -41.129476 | -71.467637 | This work. |
| Sampled | 2022 | -41.129134 | -71.449596 | This work. |
| Sampled | 2022 | -41.1276331 | -71.44916 | This work. |
| Sampled | 2022 | -41.1275 | -71.44904 | This work. |
| Sampled | 2022 | -41.1274844 | -71.448984 | This work. |
| Sampled | 2022 | -41.127265 | -71.448304 | This work. |
| Sampled | 2022 | -41.12575 | -71.447996 | This work. |
| Sampled | 2022 | -41.124952 | -71.447745 | This work. |
| Sampled | 2022 | -41.124627 | -71.447569 | This work. |
| Sampled | 2022 | -41.1246111 | -71.446887 | This work. |
| Sampled | 2022 | -41.124448 | -71.446338 | This work. |
| Sampled | 2022 | -41.1240912 | -71.4452778 | This work. |
| Sampled | 2022 | -41.1223889 | -71.445272 | This work. |
| Sampled | 2022 | -41.1221389 | -71.445225 | This work. |
| Sampled | 2022 | -41.122037 | -71.4451151 | This work. |
| Sampled | 2022 | -41.120907 | -71.442465 | This work. |
| Sampled | 2022 | -41.1206944 | -71.4391414 | This work. |
| Sampled | 2022 | -41.1205 | -71.436164 | This work. |
| Sampled | 2022 | -41.1199722 | -71.434136 | This work. |
| Sampled | 2022 | -41.119337 | -71.433971 | This work. |
| Sampled | 2022 | -41.119337 | -71.4332222 | This work. |
| Sampled | 2022 | -41.1193056 | -71.433218 | This work. |
| Sampled | 2022 | -41.1189064 | -71.4308056 | This work. |
| Sampled | 2022 | -41.118691 | -71.4277222 | This work. |
| Sampled | 2022 | -41.1183056 | -71.4268611 | This work. |
| Sampled | 2022 | -41.117655 | -71.4264722 | This work. |
| Sampled | 2022 | -41.1175556 | -71.4263611 | This work. |
| Sampled | 2022 | -41.117546 | -71.42625 | This work. |
| Sampled | 2022 | -41.117151 | -71.4262222 | This work. |
| Sampled | 2022 | -41.115322 | -71.4260556 | This work. |
| Sampled | 2022 | -41.11528 | -71.4258489 | This work. |
| Sampled | 2022 | -41.115221 | -71.4258333 | This work. |
| Sampled | 2022 | -41.115117 | -71.4254722 | This work. |
| Sampled | 2022 | -41.115064 | -71.42547 | This work. |
| Sampled | 2022 | -41.11501 | -71.4249722 | This work. |
| Sampled | 2022 | -41.1122243 | -71.4245 | This work. |
| Sampled | 2022 | -41.1063022 | -71.422869 | This work. |
| Sampled | 2022 | -41.105372 | -71.4220556 | This work. |
| Sampled | 2022 | -41.104119 | -71.4220278 | This work. |
| Sampled | 2022 | -41.102136 | -71.42175 | This work. |
| Sampled | 2022 | -41.101521 | -71.419768 | This work. |
| Sampled | 2022 | -41.099191 | -71.419265 | This work. |
| Sampled | 2022 | -41.097799 | -71.4188814 | This work. |
| Sampled | 2022 | -41.095873 | -71.418608 | This work. |
| Sampled | 2022 | -41.095649 | -71.4182778 | This work. |
| Sampled | 2022 | -41.093193 | -71.4178593 | This work. |
| Sampled | 2022 | -41.089636 | -71.41743 | This work. |
| Sampled | 2022 | -41.089062 | -71.416524 | This work. |
| Sampled | 2022 | -41.0888889 | -71.415708 | This work. |
| Sampled | 2022 | -41.087759 | -71.4143611 | This work. |
| Sampled | 2022 | -41.079202 | -71.41425 | This work. |
| Sampled | 2022 | -41.0788849 | -71.4132222 | This work. |
| Sampled | 2022 | -41.067303 | -71.413095 | This work. |
| Sampled | 2022 | -41.064561 | -71.4126944 | This work. |
| Sampled | 2022 | -41.059857 | -71.4007222 | This work. |
| Sampled | 2022 | -41.059307 | -71.40065 | This work. |
| Sampled | 2022 | -41.057716 | -71.397063 | This work. |
| Sampled | 2022 | -41.052981 | -71.391396 | This work. |
| Sampled | 2022 | -41.0517778 | -71.387979 | This work. |
| Sampled | 2022 | -41.05 | -71.387937 | This work. |
| Sampled | 2022 | -41.047006 | -71.386575 | This work. |
| Sampled | 2022 | -41.04672 | -71.385961 | This work. |
| Sampled | 2022 | -41.046111 | -71.385888 | This work. |
| Sampled | 2022 | -41.045931 | -71.379675 | This work. |
| Sampled | 2022 | -41.043039 | -71.377809 | This work. |
| Sampled | 2022 | -41.0333333 | -71.376993 | This work. |
| Sampled | 2022 | -40.95 | -71.372108 | This work. |
| Sampled | 2022 | -40.95 | -71.368611 | This work. |
| Sampled | 2022 | -40.95 | -71.367981 | This work. |
| Sampled | 2022 | -40.95 | -71.36706 | This work. |
| Sampled | 2022 | -40.95 | -71.366764 | This work. |
| Sampled | 2022 | -40.832649 | -71.3612622 | This work. |
| Sampled | 2022 | -40.781338 | -71.358911 | This work. |
| Sampled | 2022 | -40.779863 | -71.3585267 | This work. |
| Sampled | 2022 | -40.77968 | -71.340217 | This work. |
| Sampled | 2022 | -40.778487 | -71.339294 | This work. |
| Sampled | 2022 | -40.773997 | -71.328054 | This work. |
| Sampled | 2022 | -40.773261 | -71.328054 | This work. |
| Sampled | 2022 | -40.765397 | -71.315968 | This work. |
| Sampled | 2022 | -40.691807 | -71.3152601 | This work. |
| Sampled | 2022 | -40.689278 | -71.315227 | This work. |
| Sampled | 2022 | -40.686399 | -71.31 | This work. |
| Sampled | 2022 | -40.68525 | -71.269036 | This work. |
| Sampled | 2022 | -40.665297 | -71.255957 | This work. |
| Sampled | 2022 | -40.662054 | -71.229323 | This work. |
| Sampled | 2022 | -40.661753 | -71.218343 | This work. |
| Sampled | 2022 | -40.661723 | -71.188419 | This work. |
| Sampled | 2022 | -40.661298 | -71.149318 | This work. |
| Sampled | 2022 | -40.657519 | -71.148624 | This work. |
| Sampled | 2022 | -40.633667 | -71.147295 | This work. |
| Sampled | 2022 | -40.627639 | -71.651.563 | This work. |

**Bibliography**

Blackhall, M., Raffaele, E. & Veblen, T. T. Efectos combinados del fuego y el ganado en matorrales y bosques del noroeste patagónico. *Ecol. Austral* **25**, 1–10 (2015).

Brion, C*. et al.* Actualización de los Planes de Manejo de los Parques Nacionales Lanín, Nahuel Huapi, Lago Puelo y Los Alerces: Flora (Plantas Vasculares) (2013).

Dzendoletas, M. A., Cavallaro, S., Crivelli, E. & Pereyra, F. Mapa de vegetación del ejido municipal de San Carlos de Bariloche y alrededores. Río Negro. Patagonia Argentina. *Ecología* **20**, 65–88 (2006).

Ezcurra, C. & Brion, C. Plantas del Nahuel Huapi: Catálogo de la flora vascular del Parque Nacional Nahuel Huapi, Argentina. Universidad Nacional del Comahue; Red Latinoamericana de Botánica (2005).

Guerra, P. E., González, S. B. & Elbaum, J. H. Características botánicas y determinación de aceites esenciales en especies del género *Juniperus* (Cupressaceae), cultivadas en el oeste del Chubut. CIEFAP-UNPSJB. Publicación Técnica N° 33. ISSN 1514-2264 (2008).

Iglesias, A. L. Dinámica de invasión de plantas con frutos carnosos dispersadas por aves en el noroeste de la Patagonia. [Bachelor thesis. Universidad Nacional del Comahue (2015).](https://sciwheel.com/work/bibliography/14498723)

Kutschker, A. et al. (2015). Diversidad de plantas exóticas en áreas sometidas a distintos disturbios en el Parque Nacional Los Alerces, Chubut (Argentina). *Bol. Soc. Argent. Bot.* **50**, 47–59.

Martin-Albarracin, V. L., Nuñez, M. A. & Amico, G. C. Non-redundancy in seed dispersal and germination by native and introduced frugivorous birds: implications of invasive bird impact on native plant communities. *Biodivers. Conserv.* **27**, 3793–3806; 10.1007/s10531-018-1629-4 (2018).

Masciocchi, M., Villacide, J. M., Buteler, M. & Martínez, A. S. Are invasive species promoting yellowjacket invasion in Patagonia? *J. Appl. Entomol*. [**146**,](https://onlinelibrary.wiley.com/toc/14390418/2022/146/5) 547[–](https://sciwheel.com/work/bibliography/6989599)556; [10.1111/jen.12977](https://doi.org/10.1111/jen.12977) (2022).

Moguilevsky, D., Fernández, N. V., Puntieri, J. G., Outes, V. & Fontenla, S.B. Surviving after an eruption: Ecosystem dynamics and mycorrhizae in *Nothofagus pumilio* forests affected by the 2011 Puyehue Cordón-Caulle tephra. *For. Ecol. Manag*. **479**, 118535; 10.1016/j.foreco.2020.118535 (2021).

Plan de gestión del Parque Nacional Nahuel Huapi. Administración de Parques Nacionales, Argentina (2019).

Relva, M. A. & Nuñez, M. A. Factores que facilitan y retrasan la invasión de coníferas exóticas en la Isla Victoria. *Ecol. Austral* **24**, 145–153 (2014).

Rovere, A., Namiot, G., Ocampo, M. & Girscht, A. M. Planificación y gestión para la restauración en el Parque Nacional Lago Puelo. Eco gestión 2009. Primera reunión sobre planificación y legislación forestal, Chubut, Argentina (2009).

Simberloff, D., Relva, M.A. & Nuñez, M. Gringos en el bosque: introduced tree invasion in a native *Nothofagus/Austrocedrus* forest. *Biol. Invasions* **4**, 35–53; 10.1023/A:1020576408884 (2002).

Simberloff, D., Relva, M. & Nuñez, M. Introduced species and management of a *Nothofagus/Austrocedrus* forest. *Environ. Manage.* **31**, 0263–0275; 10.1007/s00267-002-2794-4 (2003).

Stecconi, M. *et al*. Desarrollo de especies leñosas nativas y exóticas en cipresales patagónicos. *Bol. Soc. Argent. Bot.* **52**, 507–522 (2017).

Toledo, C. & Kutschker, A. Plantas Medicinales en el Parque Nacional Los Alerces, Chubut, Patagonia Argentina. *Bol. Soc. Argent. Bot.* **47**, 461–470 (2012).

[Vazquez, S. M.](https://esj-journals.onlinelibrary.wiley.com/authored-by/ContribAuthorRaw/Vazquez/Miriam+Soledad), [Mariano A. Rodriguez-Cabal](https://esj-journals.onlinelibrary.wiley.com/authored-by/ContribAuthorRaw/Rodriguez%E2%80%90Cabal/Mariano+A.), M. A. & [Amico, G. C.](https://esj-journals.onlinelibrary.wiley.com/authored-by/ContribAuthorRaw/Amico/Guillermo+C.) The forest gardener: A marsupial with a key seed-dispersing role in the Patagonian temperate forest. *Ecol. Res.* 37, 270[–](https://sciwheel.com/work/bibliography/6989599)283; [10.1111/1440-1703.12289 (2021).](https://doi.org/10.1111/1440-1703.12289)
